# Taking tissue seriously means taking communities seriously

**DOI:** 10.1186/1472-6939-8-11

**Published:** 2007-10-26

**Authors:** Ross EG Upshur, James V Lavery, Paulina O Tindana

**Affiliations:** 1 University of Toronto Joint Centre for Bioethics, 88 College Street, Toronto, Ontario, M5G 1L4, CANADA; 2 Centre for Global Health Research, St. Michael's Hospital, #315, 70 Richmond Street East, Toronto, Ontario, M5C 1N8, CANADA; 3 Navrongo Health Research Centre, Ghana Health Service, PO Box 114, Navrongo, GHANA

## Abstract

**Background:**

Health research is increasingly being conducted on a global scale, particularly in the developing world to address leading causes of morbidity and mortality. While research interest has increased, building scientific capacity in the developing world has not kept pace. This often leads to the export of human tissue (defined broadly) from the developing to the developed world for analysis. These practices raise a number of important ethical issues that require attention.

**Discussion:**

In the developed world, there is great heterogeneity of regulatory practices regarding human tissues. In this paper, we outline the salient ethical issues raised by tissue exportation, review the current ethical guidelines and norms, review the literature on what is known empirically about perceptions and practices with respect to tissue exportation from the developing to the developed world, set out what needs to be known in terms of a research agenda, and outline what needs to be done immediately in terms of setting best practices. We argue that the current status of tissue exportation is ambiguous and requires clarification lest problems that have plagued the developed world occur in the context of global heath research with attendant worsening of inequities. Central to solutions to current ethical concerns entail moving beyond concern with individual level consent and embracing a robust interaction with communities engaged in research.

**Conclusion:**

Greater attention to community engagement is required to understand the diverse issues associated with tissue exportation.

## Background

Human tissues consist of a heterogeneous set of biological materials of interest from a scientific point of view for the development of diagnostic and therapeutic agents, and the study of genetic determinants of disease. Knowledge derived from human tissues research has led to fundamental advances in understanding a wide range of human diseases [1]. As clinical and basic research move to an increasingly global scope, particularly with the recent focus on tackling the major health issues in the developing world, it is important to understand the wide range of ethical issues involved in the exportation of tissue from the developing to the developed world.

Tissue analysis is a lucrative field, and it is unclear how benefits are explained to research participants, thus rendering aspects of informed consent problematic. The fate of exported tissues is often unaccounted for, making oversight difficult. Guidelines are poorly adhered to, and communities often not informed of the exportation, and when so informed, may reject such exportation and risk the loss of potential health benefits. The following cases highlight these unresolved ethical issues in the exportation of human tissues for research purposes.

Three Controversial Cases Involving Tissue Exportation from the Developing to the Developed World

### Case 1 Uganda

A newspaper reported that Uganda is losing millions of dollars due to exportation of human biological samples from the country. If this export is not stopped, researchers say, the country might no longer attract biomedical studies, which contribute a hefty amount of foreign exchange to the health sector. In the biomedical studies done, 80 per cent of human biological samples collected have been exported. Reasons for exportation are stated to be lack of storage facilities in Uganda, short supply of staff and equipment, and limited budget to stay in Uganda. The fate of specimens after exportation is unknown [2].

### Case 2 India

Indian scientists accuse foreign researchers of violating national guidelines, introduced in the late 1990s, which permitted export of tissues for research only with approval from the health ministry. The complaint is related to a study done by molecular biologists at the University of Cambridge analyzing DNA from saliva of 988 individuals in Kerala, a region with the world's highest levels of natural radiation. Indian scientists question the need for exportation of samples without approval. They fear that India's large and diverse population might serve as a source of valuable genetic information of potential commercial value. Although guidelines are in place, there is still no machinery to implement and regulate them [3].

### Case 3 Tonga

Autogen, an Australian firm, announces an agreement to collect tissue samples to study diabetes among Tongans. Autogen promises a full range of benefits to the community, including royalties for commercially successful discoveries, and provision of drugs from discoveries, free of charge. Tongans reject the offer, in part because individual consent failed to capture or reflect community values, and risked commodifying Tongan identity [4].

In this paper, we outline the salient ethical issues raised by tissue exportation, review the current ethical guidelines and norms, review the literature on what is known empirically about perceptions and practices with respect to tissue exportation from the developing to the developed world, set out what needs to be known in terms of a research agenda, and outline what needs to be done immediately in terms of setting best practices.

We argue that reliance on first person consent for tissue exportation may be insufficient and that engagement of communities from which tissues will be exported is a necessary additional requirement. This relates as much to establishing and evaluating proposed benchmarks for best practices in research in the developing world and conforming to existing guidelines, as in building trust in terms of collaboration between north and south, and in terms of developing truly legitimate, respectful and inclusive research partnerships between the developed and developing world. As well, a transparent and consistent approach to tissue exportation would have non-exploitation of vulnerable individuals and communities as its goal. It would set a minimum standard of respect for these groups to be met by all research endeavours.

## Discussion

Human tissues are collected for research purposes in clinical practice settings and in a variety of research contexts, such as genetics studies of human disease, including susceptibility to infectious diseases. Currently, there is an immense research capacity gap between developing and developed countries. Most developing countries either lack the sophisticated technology and laboratory infrastructure necessary for the analysis of human tissues, or have insufficient capacity in these technologies to permit the analyses of large numbers of samples that is now common in high-throughput screening approaches and other volume-intensive analysis methods. These approaches are increasingly common in disease contexts such as HIV/AIDS and malaria, which are major areas of research in developing countries.

There are many compelling reasons for exporting tissues. Exportation of tissue facilitates centralized analysis, allows economies of scale, and creates large datasets that can be compared and analyzed with state of the art bioinformatics analysis. Secondly, it concentrates expertise and standardizes quality. There may be tangible public health benefits resulting from this large-scale approach to science by harnessing molecular expertise in the developed world for application in the developing world. Therefore, there are many reasons to believe that the efficient exportation and centralization of the analysis of tissues can provide benefits.

On the other hand, concerns have been raised about how such practices could worsen health, and perhaps also economic, disparities between the developing and developed world [4]. Exportation fails to invest in the capacity building necessary to strengthen research in the developing world. The analysis of tissues in the same geographical and cultural milieu from which they have been taken may enhance research participation. Some low and middle-income nations are capable of developing the appropriate infrastructure to perform such analysis. Cuba, Brazil and South Africa stand as successful examples of such capacity. As has been argued by Calestous Juma, for Africa in particular, investments in technological capacity will be an important component of capacity building, and sustaining and integrating these communities into the global knowledge economy [5].

### Major Ethical Issues

Exportation of tissues raises a host of issues concerning the potential commodification and traffic of human identity, and the exploitation of communities from which tissues have been exported. Many research projects are taking place in the developing world in the context of decolonialization, where concerns about continuing domination of the developing world by former colonial masters are still relevant.

Commodification has been defined in various ways. In terms of tissue exportation, Dickenson views it as the process by which tissue acquires value such that it becomes the object of exchange. The precise value and the entire scope of potential exchange may not be evident at the time tissues are collected and exported. The exact extent to which tissues collected for research purposes can be regarded as objects of property, and who has the ultimate ownership over them remains undetermined. Charo recently drew attention to the lack of legal clarity in the developed world over governance of human tissue, showing how the dominant legal interpretations emphasize privacy interests and autonomy in decisions to donate human tissue for research purposes, effectively avoiding the issue of ownership and property rights over human tissues. The explicit recognition of such rights could have huge implications for current research uses of human tissue, possibly resulting in severe restrictions in their use [6].

But in the same way as lack of clarity on property interests currently favours relatively unrestricted use by researchers, greater clarity on the relationship between donors and their tissues may serve to enhance understanding and ensure legitimacy.

Current practices and their legal interpretation in the developed world, which reflect an almost exclusive reliance on informed consent, raise important questions about whether this approach provides an appropriate model for the developing world. This is a pressing issue in particular because of the continued advances in large-scale bioscience endeavors involving human tissue exportation. The identity value of tissues for a cultural group, as illustrated in the Tonga scenario, may not be captured in research processes that rely solely on agreed upon forms of individual consent. If the identity value of tissue matters to communities that participate in research, these interests must be given an appropriate place in determinations of whether the research is ethical.

One key issue related to the use of human tissue in international collaborative research is benefit sharing. There is potential economic benefit (as well as scientific reputation) to be derived from discoveries made from human tissues exported from the developing world. In the developed world, controversies have arisen over patents, and the considerable profits derived from research done on tissues, while those contributing the tissue were unaware of such profits and barred from sharing benefits [7]. Although legal interpretations have thus far rejected property claims to donated tissue by the donors, these interpretations do not settle the intuitive sense by many donors that there is a meaningful relationship between them and their tissues and that this relationship should figure somehow in the overall calculus of benefit sharing. Given the huge economic disparities between developed and developing world collaborators, the long history of exploitation in the context of international collaborative research, and the powerful framing effect of colonial history in much of the developing world, simply ignoring or marginalizing questions of ownership and relationship between donor and tissue may have higher costs to the research enterprise than has been the case in developed countries.

There has been an emerging perspective in the developed world that one-time consent with future uses approved by legitimate research ethics boards (REBs) is the best model for managing tissue donations in research [8]. Whereas informed first person consent for use and reuse of tissues has become the standard in the developed world, the model may not be adequate in the developing world context. Research on tissue samples in the developing world, specifically genetic research, is now commonly conducted in conjunction with large-scale epidemiological research programs. These programs go far beyond small clinical research projects and directly implicate communities and whole populations. Given what we know about the potential for communities to suffer harm as a result in their participation in research [9] and their ability to appropriately and forcefully express their concerns about inadequate reflection of their interests in benefit sharing agreements [10], it seems inescapable that the prevailing model of individual consent for the use of tissue also has implications for communities. Many of these communities can be regarded as vulnerable and relatively disempowered. How communities should be engaged in deliberations about tissue exportation, and under what terms and conditions, remains an unanswered question.

### Current Ethical Guidelines

Current guidelines only partially address the issues, as for the most part guidance has been created for application in the developed world. These documents assume that tissues will be analyzed locally, and therefore are silent on establishing criteria for legitimate exportation. Guidelines from the developing world are similarly silent on the need to engage communities or justify exportation. Guidelines from both contexts universally endorse the need for informed consent from participants, but are, for the most part, unclear on the necessity of documenting and justifying exportation, or of explaining the potential cultural significance of tissue exportation.

Recently published ethical benchmarks for research in the developing world establish a set of frameworks that can be used to evaluate progress towards best practices in tissue exportation. For example, Emanuel *et al*. have articulated a set of benchmarks for research ethics in the developing world [11]. As well as expanding concerns for the assurance of the cultural appropriateness of informed consent, there are a series of benchmarks that call for collaborative partnerships as necessary to the ethical justification of research in the developing world.

These benchmarks are broadly relevant to tissue exportation. They call for engagement in partnership with national and/or international research institutions. They recommend collaboration with local and national researchers, health policymakers and the community. They should share responsibilities for determining the importance of health problems, assessing the value of the research, and for planning, conducting, and overseeing the research, and integrating the research into the health system. They call for respecting the community's values, culture, traditions and social practices. They suggest contributing to capacity development for researchers and health policy-makers, so the community can become a full and equal partner in the research enterprise. They say recruited participants and communities should receive benefits from the conduct and results of research, sharing fairly in the financial and other rewards of the research.

These benchmarks do not speak directly to determining when tissues can and should legitimately be exported, or of explaining the meaning and significance of exportation as part of the process of consent. Importantly, the benchmarks do not necessitate engagement with the community on issues of identity that are entailed by the analysis of tissues. These may, in fact, be the principal justification for privileging local perspectives and values. The risk of exploitation increases if identity concerns are not adequately accounted for, as it may be harder, even impossible, to ensure truly fair distribution of benefits if such perspectives are not known.

The common practice of stripping samples of identifiers, which creates conditions of anonymity and renders concerns for consent moot under most guidelines, is not a constructive response to calls for increased attention to identify issues related to the use of human tissue in research. Once stripped of identifiers and exported from the context where they have been collected, tissues lose most of their scientific value, particularly in the context of epidemiological research. The potential for meaningful benefits that might be shared equitably is therefore dramatically undermined, or eliminated entirely. The protection of identity, and the recognition of a significant relationship between donors and their tissues, even if this has not been formally recognized by Western courts as a formal property right, should be viewed as strong community interests, and should be treated as such in guidelines on these issues, and should be incorporated explicitly into the negotiations and deliberations that establish the tissue collection practices within any collaborative research project.

### What is Known

While there is a growing body of scholarship on the perspectives of research subjects regarding the use of their tissues in the developed world, little is known about the perspectives of research subjects in the developing world. The extent to which international guidelines and proposed international benchmarks are recognized, comprehended, or adhered to by study participants, study communities, researchers and ethics review boards is, for the most part, unknown. Recent empirical studies indicate a mixed picture, with significant gaps in understanding.

A recent study in Kenya showed that a spectrum of views exists, with a considerable complexity of perspectives concerning understanding the use of tissues in research [12]. There is confusion in the eyes of many participants about the precise distinction between the need for tissue for research and for clinical care. More specifically, there is confusion about what blood and tissue will be used for, and concern about why it is necessary to export such tissue for research purposes. Respondents reported varying interest in being provided with the results from future studies using their stored samples. Similar findings have also been found in the developed world context.

Wendler *et al*. surveyed participants in a malaria clinical trial in Uganda and reported that most participants were willing to permit samples to be stored and exported, and were willing to waive additional consent for subsequent research, provided the study was approved by an ethics review board [13]. Respondents also were interested in knowing what sorts of studies stored samples would be used in, suggesting that their expressions of willingness to donate samples for storage and export were not exhaustive of their interests in the fate of their tissues. But the study did not ask respondents about the deeper issues of community identity, and whether this was of concern to them and, if so, how such concerns might be addressed in the context of the research collaboration.

Langat, in a study of two Kenyan ethics review boards, found that 25 per cent of protocols reviewed stated there was a need for tissue storage and reuse, but only half actually informed participants of this. He concluded that investigators "do not see the need to seek consent for storage reuse and exportation of samples [14]."

The empirical literature points to the urgent need for further research. If Wendler's findings are representative, the role of REBs would be crucial in ensuring the legitimate use of stored and exported samples. Langat's results, though, are cautionary. Clearly if REBs are to be the locus of approval for research on stored samples, there is a pressing need for the creation of local REBs, and expanded education of existing REBs on the full spectrum of ethical issues associated with tissues research. REBs require the capacity to monitor and audit studies to ensure not only that appropriate informed consent has been obtained, but that the agreed upon practices are followed by investigators. They need to ensure that communities have been consulted. While there is currently no consensus on specific mechanisms for community consent, the practice of eliciting broader community authorization may have a role to play in contexts where tissues will be exported from the community, particularly in those situations where the analysis has implications for more than the consenting individual.

Additionally, REB oversight on exported tissue becomes problematic and to all intents and purposes non-existent once the tissue has left the context from which it was obtained. Some mechanism must be in place to account for the fate of the tissues, and innovative ways of REB collaboration between North and South may be required to aid in this oversight process.

### What Needs to be Known

There is a need for more studies such as those described above, in diverse parts of the developing world. They should examine the extent to which tissue exportation is consented to, and the extent to which communities that are being researched understand the rationales for tissue exportation and storage. There are no studies investigating researchers' perceptions on the need for consent, or their awareness and perspectives on guidelines. Similarly, there have been no studies that look specifically at the perceived relationship between donors and their tissues and the significance of the relationship for communities. One case study from Malawi about the use of tissue taken from the eyes of deceased children during autopsy-based research on causes of death from cerebral malaria [15] reveals that tissues can have profound cultural significance, in this case raising fears that researchers might use the tissues to exercise supernatural powers over the deceased child and thereby disrupt prospects for a peaceful afterlife. To avoid previous colonial errors, such cases should not be dismissed as quaint by Western researchers and REBs, but rather should encourage more and better research into the various ways in which tissue might be imbued with rich and powerful cultural meaning and how this meaning might give rise to ethically significant interests on the part of participating communities. To get at the root of these interests it will be necessary to go beyond survey designs, which pose questions from the perspective of the researchers, to more naturalistic modes of enquiry, which can help to reveal insights and meaning that survey methods can't reach [16].

There is a research agenda that should focus on broad community engagement, including a sustained investigation of the cultural acceptability of the exportation and storage of tissues for research purposes. It is important to include investigators and REBs in the concept of community engagement in the research enterprise, as the need to harmonize expectations among all stakeholders is clearly shown by the brief but informative empirical literature. A series of comparative case studies looking at practices and perceptions across several developing world contexts would be a logical first step to informing the process.

There is also a need to address, particularly as research and databases merge into large-scale research programs, how best to deal with future unforeseen uses of stored exported tissues, and to consider innovative means of engaging research participants that can enhance local control over data [16]. Similarly, what constitutes fair distribution of research benefits in the context of international collaborative research, and which model is best for addressing issues of ownership or relationship between tissue donors and their exported tissue, requires further elucidation, including an expansion of the notion of benefits to include the value of maintaining oversight control of valuable cultural property, such as genetic heritage.

## Conclusion

Ensuring ethical research entails bringing communities together in dialogue. These communities include researchers, the researched and those who will provide oversight, such as REBs.

At minimum, there is a need to ensure documentation of explicit and legitimate consent for the exportation of human tissue for research purposes. Legitimate consent should include engagement with communities with respect to the necessity for tissue exportation, as well as agreed upon and explicit standards for current use, and a process to manage future uses of exported tissue. The length of time that such tissues will be retained, who has access to the tissue, and all potential commercial benefits from the tissues should be included in the documentation. Particular attention to understanding perceptions and expectations on possible future uses of tissues is needed. As well, models of REB accountability for exported tissues are required and may need innovative solutions. It is incumbent upon the investigators to explain the reasons why capacity building locally is not necessarily a full alternative to the need for exportation. Provisions such as these will help build trust and ensure transparency in communications, and will also help to create an understanding of the cultural and social concerns with tissue exportation.

However, satisfying the conditions of individual consent is not sufficient to ensure that ethical standards have been met. Researchers must ensure that deeper accounts of the meaning and value of tissues as culturally meaningful artifacts are explicitly identified and incorporated into deliberations about appropriate use and exportation of tissues. Concerns about the fair and respectful treatment of exported tissues should also figure more prominently in research ethics review, including developing appropriate guidance about their fate and ultimate disposition.

## Competing interests

The author(s) declare that they have no competing interests.

## Authors' contributions

RU conceived the paper and wrote the first draft. PT and JL contributed equally to the revision of the manuscript. All authors read and approved the final manuscript.

## Pre-publication history

The pre-publication history for this paper can be accessed here:


